# Establishment of Reference Intervals for Thrombus Biomarkers in a Single‐Center Chinese Population Using Indirect Methods

**DOI:** 10.1002/jcla.70315

**Published:** 2026-09-09

**Authors:** Lei Zhang, Qiyu Hu, Yiming Chen, Hui Yuan

**Affiliations:** ^1^ Department of Clinical Laboratory Beijing Anzhen Hospital, Capital Medical University Beijing China; ^2^ Department of Clinical Laboratory Beijing Jishuitan Hospital, Capital Medical University Beijing China

**Keywords:** indirect method, PIC, reference interval, TAT, TM, t‐PAIC

## Abstract

**Background:**

Thrombin‐antithrombin complex (TAT), plasmin‐α2‐antiplasmin complex (PIC), thrombomodulin (TM), and tissue plasminogen activator‐inhibitor complex (t‐PAIC) are key biomarkers reflecting coagulation/fibrinolysis activation and endothelial injury. Population‐specific reference intervals (RIs) are essential for accurate diagnosis, but Chinese‐specific RIs remain limited. This study aimed to establish reliable RIs for these four markers in a Chinese population using indirect methods applied to clinical big data.

**Methods:**

Anonymized clinical data for TAT, PIC, TM, and t‐PAIC from 2020 to 2024 at Beijing Anzhen Hospital, Capital Medical University, were collected. After data reduction, deduplication, and outlier removal, RIs were calculated for the overall population and stratified by sex and age using indirect methods (Hoffmann and Bhattacharya). Reliability was verified by comparing the derived RIs with manufacturer‐provided ranges using reference change value (RCV) criteria.

**Results:**

Overall RIs determined by the Hoffmann method for TAT, PIC, TM, and t‐PAIC were 0.64–3.57 ng/mL, 0.25–1.08 μg/mL, 5.64–13.42 TU/mL, and 3.22–15.88 ng/mL, respectively. Those determined by the Bhattacharya method were 0.58–2.84 ng/mL, 0.25–1.11 μg/mL, 5.60–14.53 TU/mL, and 3.46–15.44 ng/mL, respectively. TAT and PIC levels were higher in females, whereas TM and t‐PAIC were higher in males. All four markers increased with age. Relative differences between method‐derived RIs and manufacturer ranges were within RCV limits.

**Conclusion:**

Both Hoffmann and Bhattacharya's indirect methods are reliable for establishing RIs for TAT, PIC, TM, and t‐PAIC. The study provides population‐specific RIs for a Chinese cohort, supporting accurate early detection and risk assessment of thrombotic diseases.

## Graphical abstract

This study established and validated comprehensive RIs for four key thrombus biomarkers (TAT, PIC, TM, and t‐PAIC) in a Chinese population using the Hoffmann and Bhattacharya indirect methods on a large clinical dataset. The tailored RIs offer a practical tool for early detection and risk assessment of thrombotic diseases, supporting more precise and personalized clinical decision‐making.

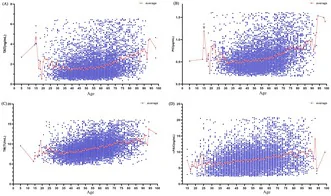

## 1 Introduction

Thrombotic diseases pose a significant global threat. Venous thromboembolism (VTE), encompassing deep vein thrombosis (DVT) and pulmonary embolism (PE), is the third leading cause of cardiovascular disease and contributes substantially to global mortality, disability, and healthcare burden [1, 2]. In China, VTE is a growing concern, with studies indicating high incidence and mortality rates among hospitalized patients [3]. A recent nationwide study estimated over 200,000 PE cases in China in 2021, with an incidence rate of 14.19 per 100,000, approaching Western levels and challenging prior perception of low VTE incidence in East Asia [4]. These findings underscore the urgent need for early diagnosis and intervention.

Laboratory biomarkers are essential for diagnosing and managing thrombotic diseases. Traditional coagulation tests like prothrombin time (PT), activated partial thromboplastin time (APTT), and fibrinogen (FIB) mainly assess bleeding risk and lack sensitivity for early thrombosis, thereby limiting early diagnosis. d‐dimer and fibrin(ogen) degradation products (FDPs), which result from thrombus breakdown, indicate fibrinolytic activation but lag behind the initial coagulation phase [5]. In recent years, four novel thrombus markers—thrombin‐antithrombin complex (TAT), plasmin‐α2‐antiplasmin complex (PIC), thrombomodulin (TM), and tissue plasminogen activator‐inhibitor complex (t‐PAIC)—have emerged for earlier detection of thrombus formation [6, 7]. TAT, a 1:1 complex of thrombin and antithrombin III, has a short half‐life (3–15 min) and serves as a molecular marker of thrombin generation, with elevated levels suggesting hypercoagulability during anticoagulant therapy [8]. PIC, with a half‐life of approximately 6 h, reflects plasmin generation and aids in diagnosing and monitoring fibrinolytic disorders [9]. TM, primarily produced by endothelial cells, acts as a receptor and cofactor for thrombin, and increased plasma TM concentration indicates endothelial cell injury and reduced anticoagulant capacity [10]. t‐PAIC is a 1:1 complex formed between tissue‐type plasminogen activator (t‐PA) and its physiological inhibitor, plasminogen activator inhibitor‐1 (PAI‐1), which positively correlates with t‐PA concentration and the degree of vascular endothelial injury, serving as a direct marker for assessing endothelial damage and fibrinolytic activation [11]. Combined detection of these four markers enables comprehensive assessment of coagulation and fibrinolytic activation, as well as vascular endothelial injury, with significant clinical value for early diagnosis of disseminated intravascular coagulation (DIC), thrombotic diseases, and monitoring of thrombolytic efficacy [12]. Zhou et al. demonstrated that this marker panel is an effective diagnostic and prognostic tool for VTE in cancer patients [13].

Reference intervals (RIs) are fundamental in medical decision‐making and directly affect diagnostic accuracy, treatment appropriateness, and prognostic reliability [14]. However, RIs vary with demographic characteristics (e.g., age, sex, ethnicity), geographical environment, diet, and genetic background [15]. The Chinese population has distinct genetic and lifestyle characteristics, making RIs derived from Western populations potentially inappropriate. Many healthcare institutions still rely on manufacturer‐provided RIs, often based on data from Western populations or small‐sample studies, which may introduce diagnostic bias [16]. Therefore, establishing population‐specific RIs is crucial. RIs can be established using direct and indirect methods [17]. The direct method recruits healthy volunteers and, although considered ideal, is time‐ and resource‐intensive and may introduce selection bias due to stringent inclusion and exclusion criteria that do not fully represent the target population [18]. In contrast, the indirect method utilizes large volumes of clinical data generated during routine medical care and applies statistical modeling to derive RIs [19]. This approach avoids additional recruitment, reduces cost, leverages large existing datasets, and better reflects real‐world clinical conditions [20]. Among indirect approaches, the Hoffmann [21] and Bhattacharya [22] methods are most commonly used. Studies show that indirect RIs generally agree well with direct ones and more accurately represent local physiological characteristics [23, 24, 25].

Therefore, this study utilized indirect methods and large‐sample clinical data from a Chinese population to establish RIs for TAT, PIC, TM, and t‐PAIC. The outcomes will provide clinical laboratories with accurate, population‐specific RIs to support earlier diagnosis and improved risk assessment of thrombotic diseases [16, 26], ultimately contributing to better patient outcomes and reduced clinical and socioeconomic burden.

## 2 Materials and Methods

### 2.1 Materials

Data for all tests of the four thrombus markers (TAT, PIC, TM, and t‐PAIC) stored in the Laboratory Information System (LIS) of Beijing Anzhen Hospital, Capital Medical University, from 2020 to 2024 were extracted and screened for analysis. Exclusion criteria were: (1) missing name or age, or data from foreign nationals; (2) age recorded in days, weeks, or months; (3) multiple measurements from the same patient, for which only the first result was retained; and (4) test results containing “<” or “>”.

After applying these criteria, data were log‐transformed to approximate normality and subjected to iterative outlier removal using Tukey's method, where values beyond P25–1.5 × interquartile range (IQR) and P75 + 1.5 × IQR were removed until no extreme outliers remained.

### 2.2 Instruments and Reagents

Detection was performed using a fully automated HISCL‐800 chemiluminescence immunoassay analyzer (Sysmex, Kobe, Japan) with matched reagents, calibrators, and quality controls from the same manufacturer. The instrument underwent routine daily calibration and maintenance according to manufacturer instructions. Daily quality control ensured testing precision, and all procedures followed standard operating protocols. Within‐run and between‐run coefficients of variation (CVs) for all four markers were maintained below 10%. Detailed batch information is provided in Table S1.

### 2.3 Establishment of RIs and Statistical Analysis

Statistical analyses were performed using SPSS 26.0. The Kolmogorov–Smirnov test was used to assess normality. Normally distributed variables are expressed as mean ± standard deviation ($\bar{x}$ ± SD), and non‐normally distributed data as median (*M*) with interquartile range (P25, P75). After data processing described in Section 2.1, datasets were used for further analysis. Data processing was performed using Microsoft Excel 2021. RIs for the four thrombus markers were calculated for the overall population and stratified by sex and age groups using the Hoffmann and Bhattacharya methods. The Hoffmann theoretical quantile curve and the Bhattacharya log‐frequency plot were used to identify the linear range for determining RI limits. Procedures for the Hoffmann method followed references [21, 24]. Briefly, test results were entered into an MS Excel spreadsheet, log‐transformed, sorted in ascending order, and a distribution series was generated using the Excel function NORM.INV. A quantile‐quantile (Q–Q) plot was constructed, and the linear segment was identified. *Y* values at *X* = −2 and *X* = 2 were calculated from the regression equation. Under the standard normal distribution, the interval *μ* ± 2*σ* represents the central 95% range (2.5th–97.5th percentile), that is, the RI (*μ* denotes the mean and σ the standard deviation). According to the Hoffmann method, P5 and P95 of the linear segment were considered the RI limits. Differences between sexes were assessed using independent‐samples *t*‐tests, and differences among age groups using one‐way analysis of variance (ANOVA). *p* < 0.05 was considered statistically significant.

### 2.4 Verification of RIs

To assess the reliability and clinical applicability of the indirect method‐derived RIs, two verification approaches were used.

First, RIs established by the Hoffmann and Bhattacharya methods were compared with those provided in the manufacturer's reagent inserts. Agreement was assessed by calculating relative differences between the upper and lower limits derived from indirect methods and manufacturer values. These differences were compared with the reference change value (RCV). Let RI_upper and RI_lower denote the upper and lower limits of the RI established in this study, and upper reference limit (URL) and lower reference limit (LRL) denote the manufacturer limits. The relative difference for the upper limit was calculated as:

(1) $$\text{Relative difference}\;\left(\text{upper}\right)=\left(\mathrm{RI}\_\text{upper}-\mathrm{URL}\right)/\mathrm{URL}\times 100\%$$

For the lower limit, calculation depended on whether LRL was zero. For TAT, PIC, and t‐PAIC, the manufacturer's LRL is 0, which lacks clinical interpretability as a denominator; therefore, the URL was used instead:

(2) $$\text{Relative difference}\;\left(\text{lower},\mathrm{LRL}=0\right)=\left(\mathrm{RI}\_\text{lower}-\mathrm{LRL}\right)/\mathrm{URL}\times 100\%$$

For TM, the manufacturer's LRL is a non‐zero clinically meaningful value, so the LRL itself served as the denominator:

(3) $$\text{Relative difference}\;\left(\text{lower},\mathrm{LRL}\neq 0\right)=\left(\mathrm{RI}\_\text{lower}-\mathrm{LRL}\right)/\mathrm{LRL}\times 100\%$$

The RCV, which defines the minimal clinically significant change, was calculated as follows:

(4) $$\mathrm{RCV}=\surd 2\times Z\times \surd \left({{\mathrm{CV}}_{a}}^{2}+\mathrm{CV}i^{2}\right)$$

(5) $${\mathrm{CV}}_{a}=\surd \left({\text{CV1}}^{2}+{\text{CV2}}^{2}\right)$$

In Formula (4), *Z* = 1.96 (95% confidence level), CV_a_ represents analytical imprecision and is calculated from CV1 and CV2 of two levels of quality control materials using Formula (5). CV_i_ represents within‐subject biological variation obtained from a public database [27]. If both upper and lower relative differences were less than the RCV, agreement was considered acceptable.

Second, following CLSI EP28‐A3c recommendations for verifying age‐ and sex‐partitioned RIs, for each reference subgroup (i.e., each sex or age group for which a RI was established), 20 healthy individuals undergoing health examinations at our institution from May to October 2024 were selected as reference subjects. Their results were compared with established RIs. An RI was considered suitable for the local population if ≤ 10% of values fell outside the interval. Verification pass rate was calculated as: (number of individuals with test values within the RI/20) × 100%.

## 3 Results

### 3.1 Data Distribution Characteristics for Indirect RI Establishment

**FIGURE 1:**
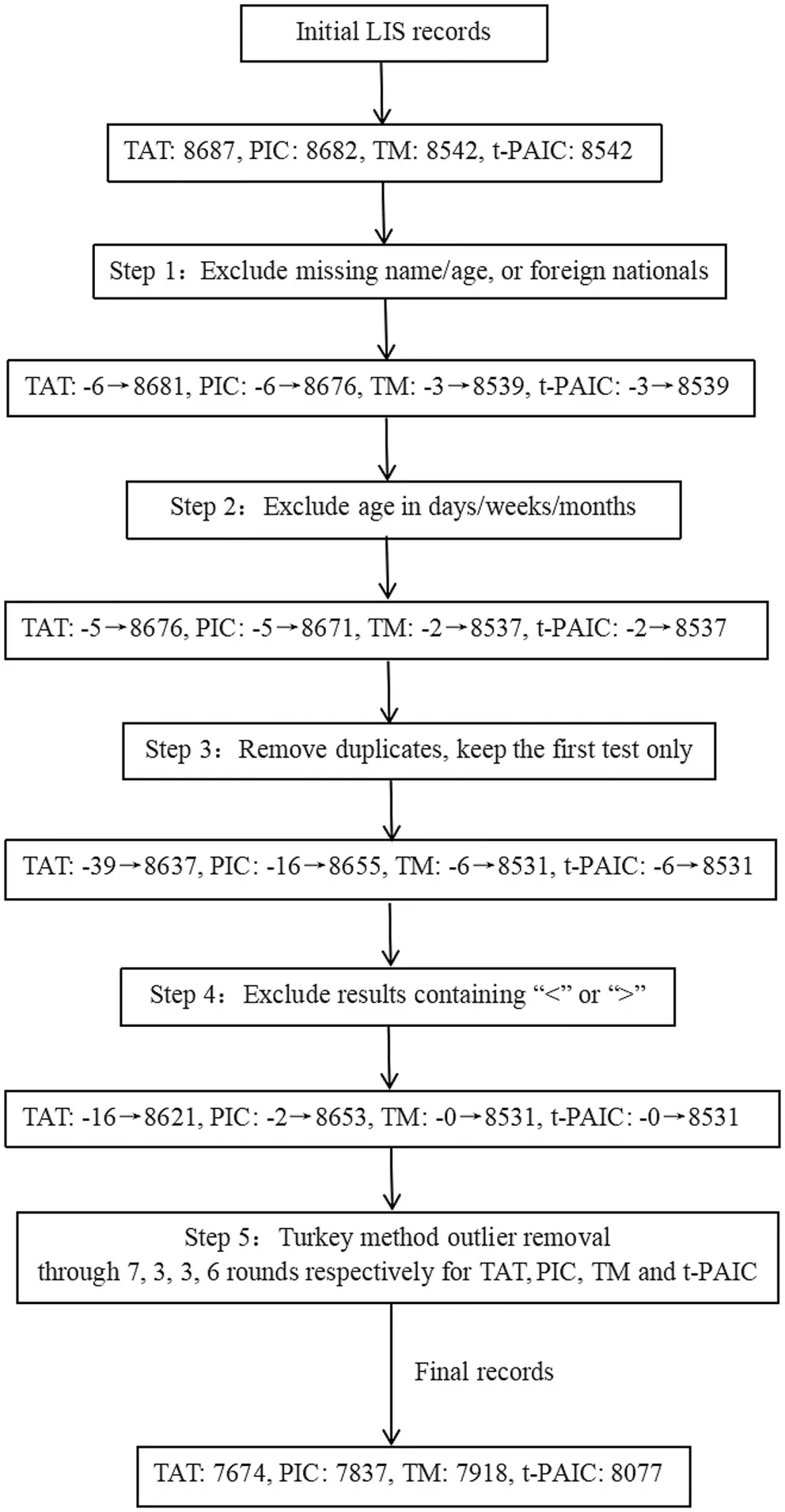
Flowchart of data selection for TAT, PIC, TM, and t‐PAIC.

**FIGURE 2:**
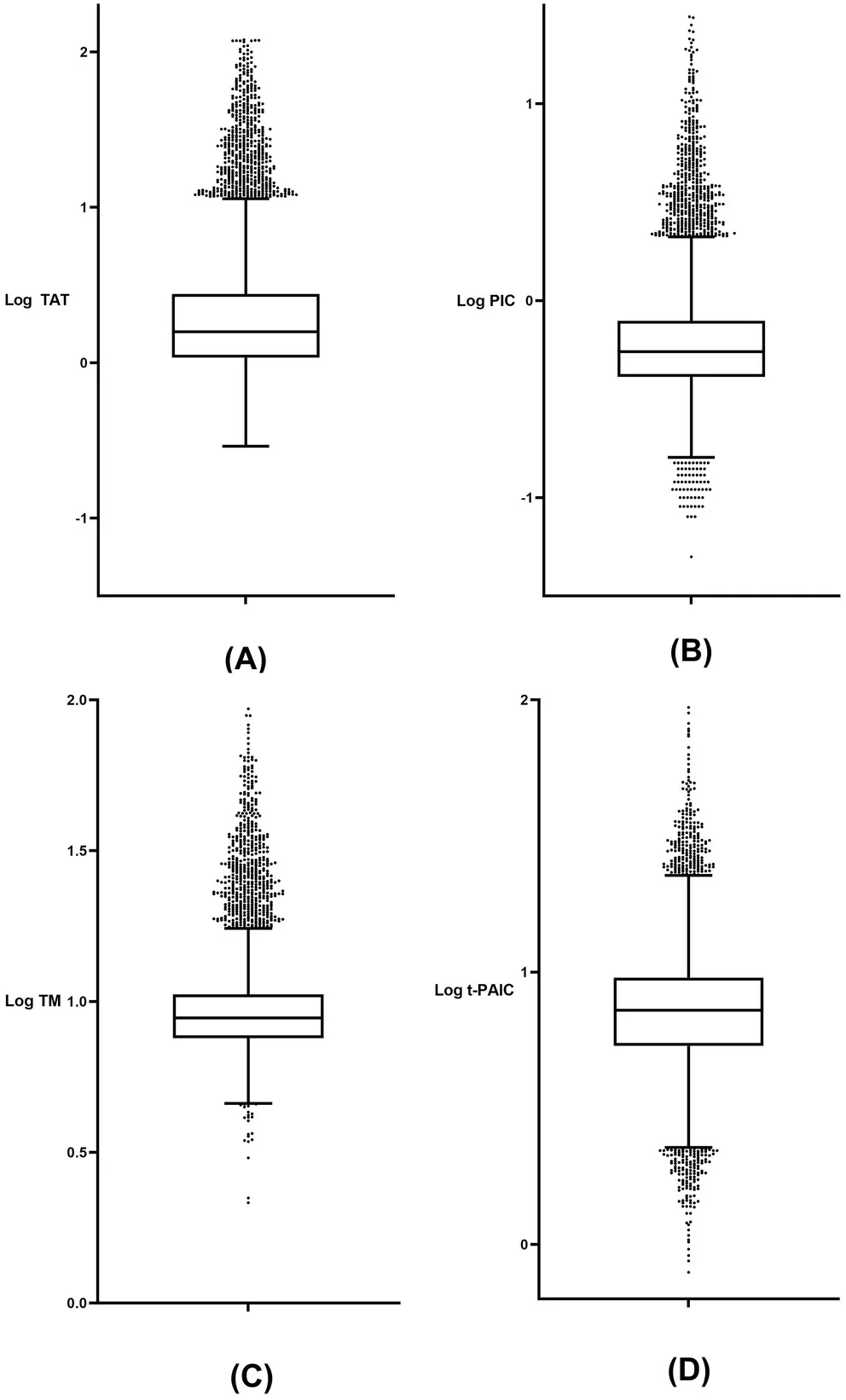
Box plots of log‐transformed thrombus marker data. Panels (A–D) show log‐transformed levels of TAT, PIC, TM, and t‐PAIC, respectively. Each box represents the interquartile range (IQR; P25–P75), the horizontal line inside the box indicates the median, and whiskers extend to values within 1.5 × IQR from the lower and upper quartiles. Black dots below or above the whiskers indicate outliers.

The initial records retrieved from the LIS were 8687 for TAT, 8682 for PIC, 8542 for TM, and 8542 for t‐PAIC. After applying the exclusion criteria (missing name/age/foreign nationals, age in days/weeks/months, duplicate patients, and results containing “<” or “>”), the remaining numbers were 8621, 8653, 8531, and 8531, respectively. All four markers exhibited skewed distributions. After log transformation, outliers were iteratively removed using Tukey's method until no extreme values remained. This process required seven, three, three, and six screening rounds for TAT, PIC, TM, and t‐PAIC, respectively, reflecting differences in their initial distributions. Following this cleaning, 7674, 7837, 7918, and 8077 effective results were retained for analysis. Detailed data selection steps and remaining records at each stage are illustrated in Figure 1. The above cleaned data approximated normal distributions after log transformation. Figure 2 presents box plots of the log‐transformed values for the four markers.

### 3.2 Quality Control of the Detection System

Quality control was performed according to performance verification requirements for quantitative testing in clinical hematology laboratories. Imprecision was assessed using the cumulative coefficient of variation (CV). Two concentration levels of quality control materials were used for long‐term internal quality control of each marker, consistent with routine daily QC performed throughout the study period, and all performance indicators met the required standards. Internal quality control followed Westgard multi‐rules (1–3 s, 2–2 s, R‐4 s). Both levels of quality control were tested daily before sample analysis, and testing proceeded only when control results were acceptable, ensuring that all data were obtained under stable internal quality control conditions. The laboratory passed the National Centre for Clinical Laboratories external quality assessment program in 2024.

The calculated CV_*a*_, CV_*i*_, and RCV values for the four markers are shown in Table 1, where CV1 and CV2 represent the annual cumulative CVs for the two levels of internal quality control materials for each marker in 2024, calculated from 331 individual quality control measurements per level collected throughout the year on days when clinical samples were tested for these markers.

**TABLE 1:** Imprecision and RCV values for TAT, PIC, TM, and t‐PAIC.

Parameter	TAT (%)	PIC (%)	TM (%)	t‐PAIC (%)
CV1 (%)^a^	3.23	6.78	5.77	7.69
CV2 (%)^b^	4.81	7.73	6.31	8.03
CV_a_ (%)^c^	5.79	10.28	8.55	11.11
CV_i_ (%)^d^	26	11.4	18.0	15.9
RCV (%)	73.84	42.55	55.24	53.79

Abbreviations: CV, coefficient of variation; PIC, plasmin α2‐antiplasmin complex; RCV, reference change value; TAT, thrombin–antithrombin complex; TM, thrombomodulin; t‐PAIC, tissue‐type plasminogen–activator‐inhibitor complex.

^a^ Cumulative CV for level‐1 quality control material (2024).

^b^ Cumulative CV for level‐2 quality control material (2024).

^c^ Analytical imprecision calculated from CV1 and CV2.

^d^ Within‐subject biological variation, obtained from an online database [27].

### 3.3 Establishment of RIs

**FIGURE 3:**
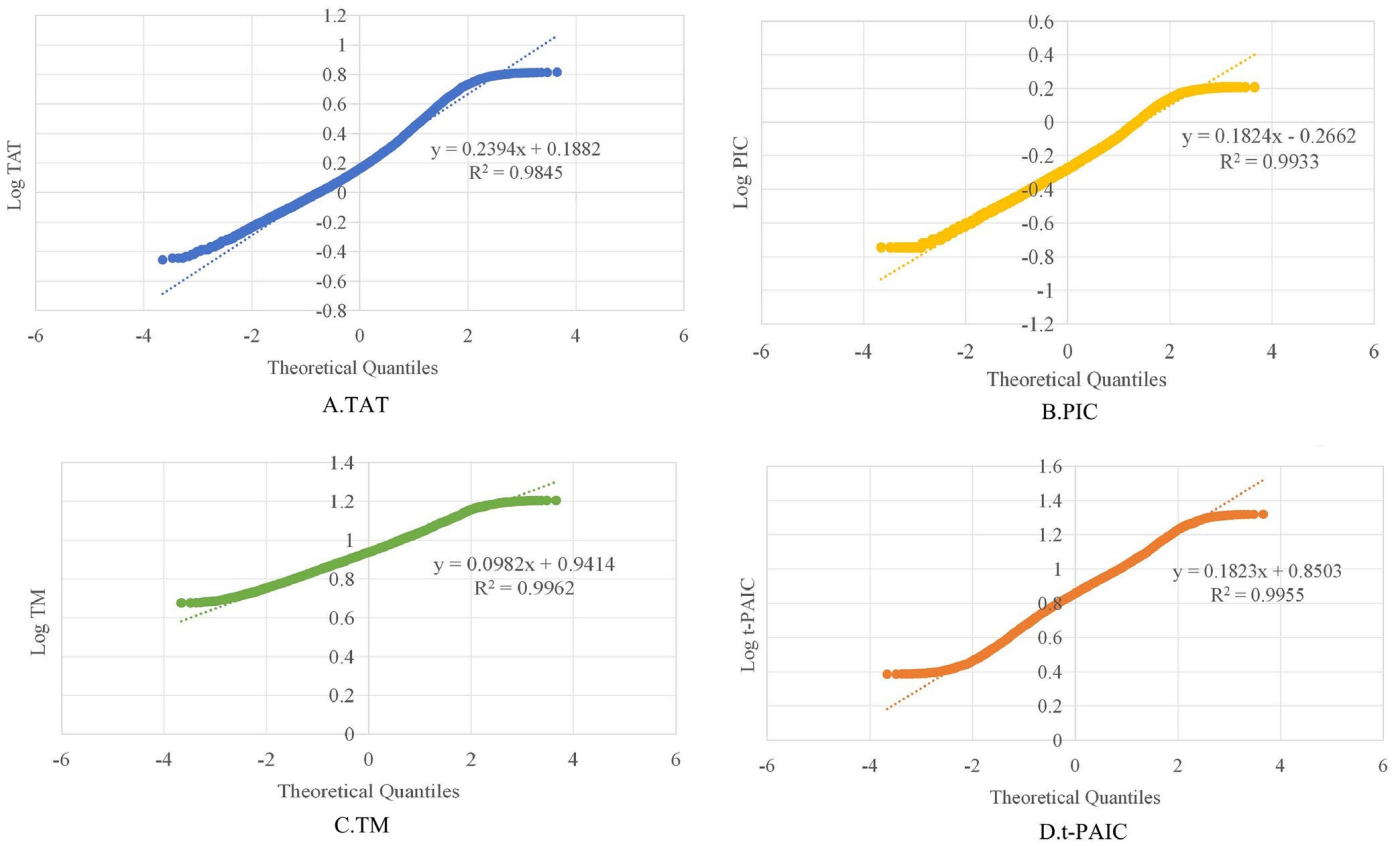
RI calculation using the Hoffmann method. Panels (A–D) correspond to TAT, PIC, TM, and t‐PAIC. For each biomarker, the theoretical quantile plot and fitted regression line are shown for identifying the linear segment used to derive the RI limits.

**FIGURE 4:**
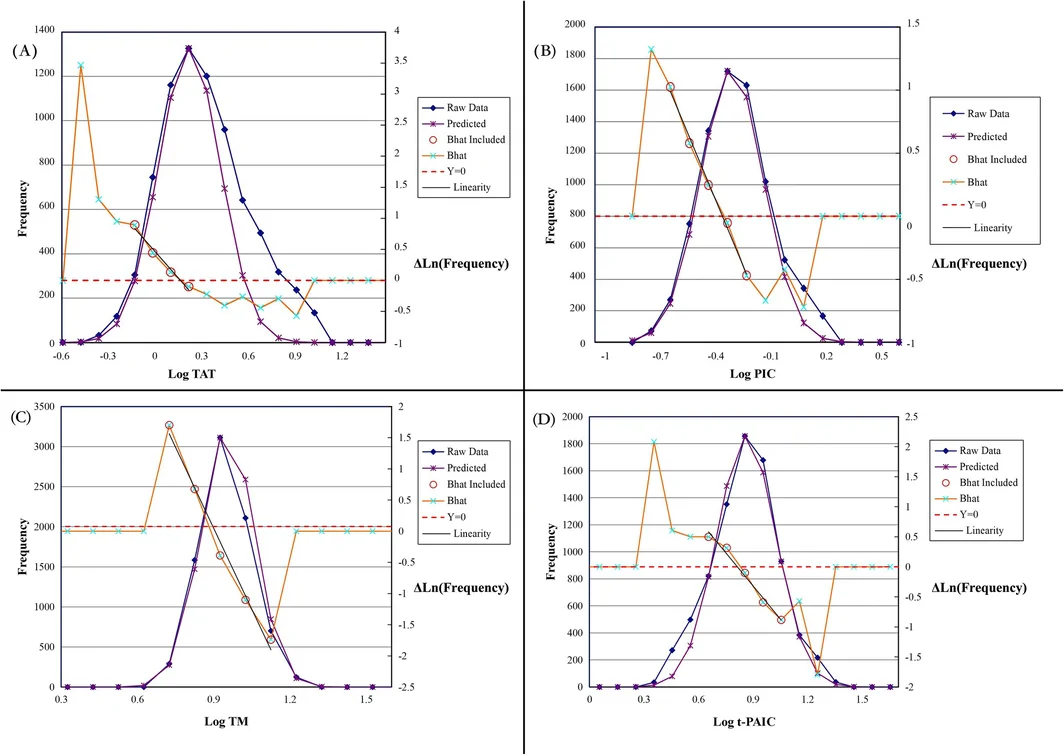
RI calculation using the Bhattacharya method. Panels (A–D) show the frequency distribution curves for TAT, PIC, TM, and t‐PAIC after log transformation. The smoothed frequency plot and the Bhattacharya linear portions used for RI estimation are illustrated.

**FIGURE 5:**
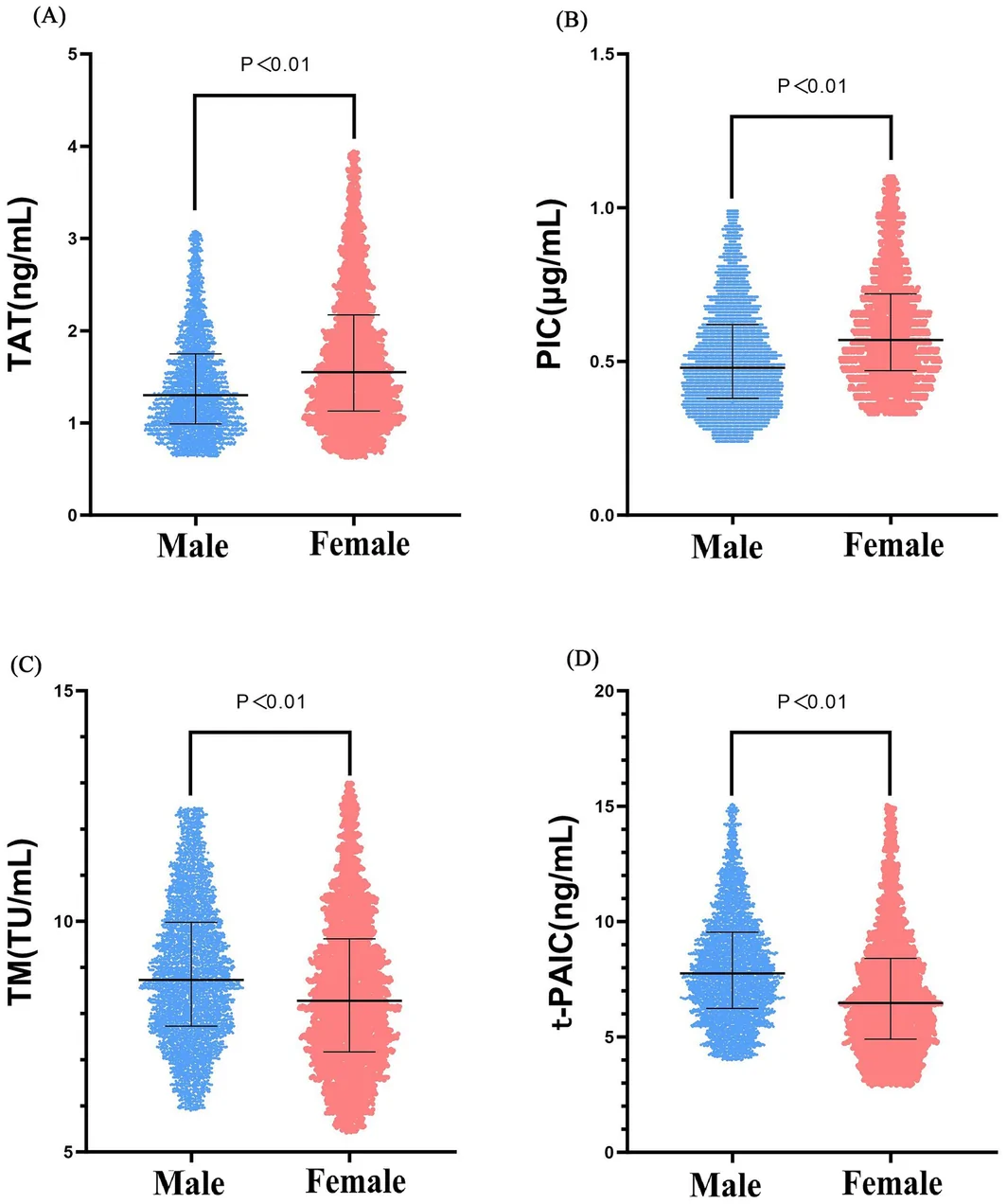
Sex‐based comparison of thrombus markers. Panels (A–D) display TAT, PIC, TM, and t‐PAIC levels in males and females. Bold horizontal lines indicate medians and interquartile ranges. *p*‐values represent the comparison between sexes.

**FIGURE 6:**
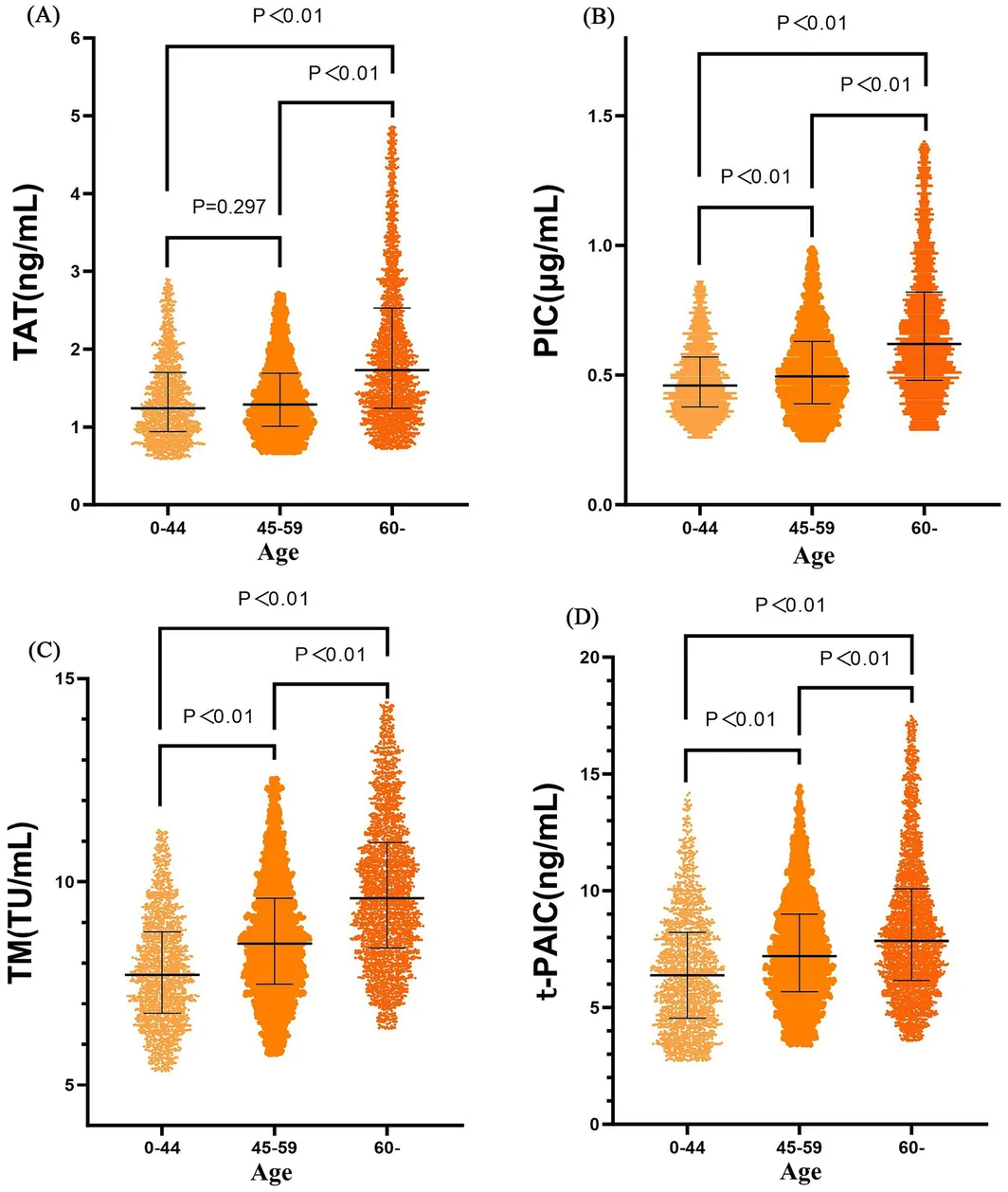
Age‐group differences in thrombus markers. Panels (A–D) present TAT, PIC, TM, and t‐PAIC across the age groups 0–44, 45–59, and ≥ 60 years. Horizontal lines show medians and interquartile ranges. Significant between‐group comparisons are annotated with *p*‐values.

**FIGURE 7:**
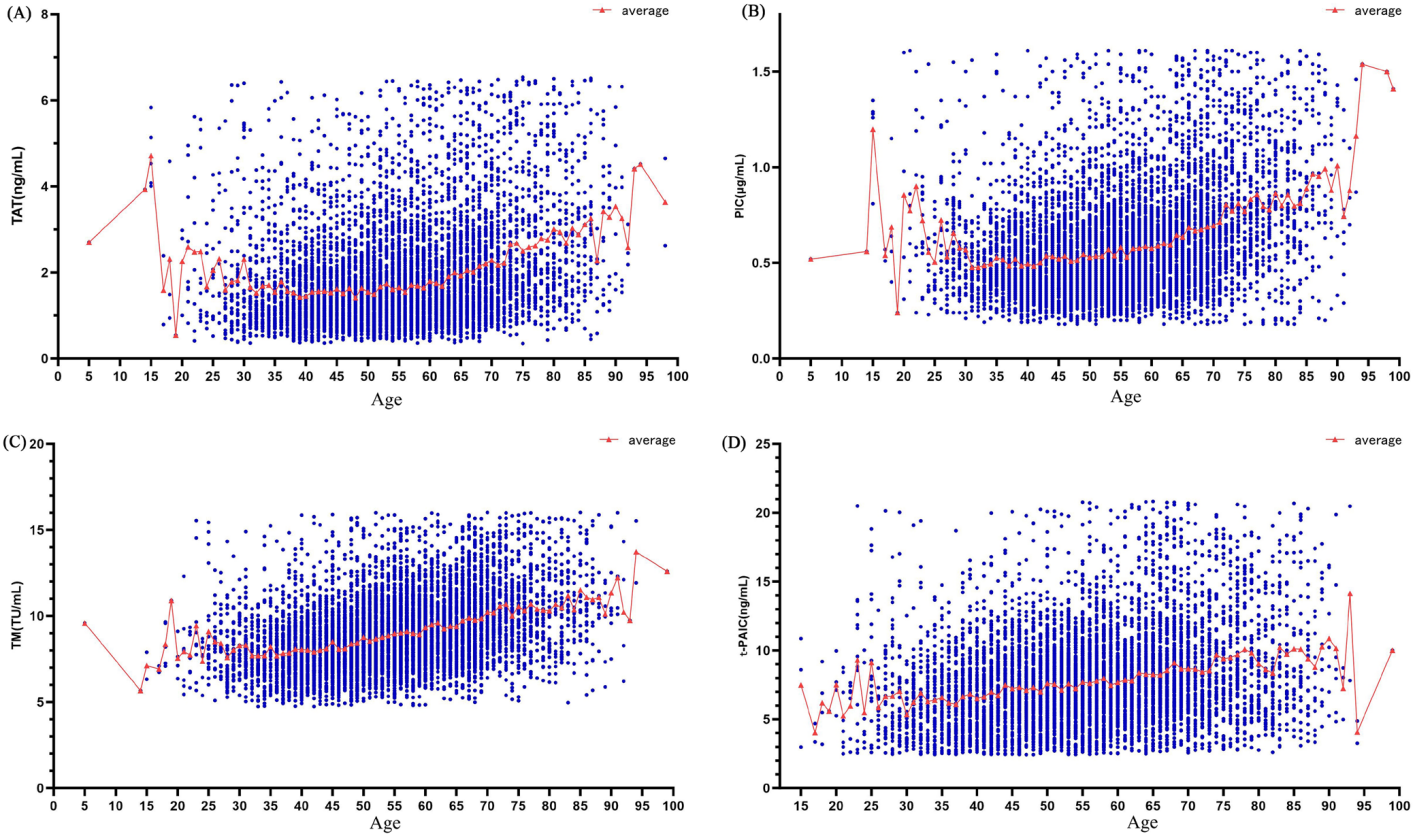
Scatter distribution of thrombus markers by age. Panels (A–D) (TAT, PIC, TM, t‐PAIC) plot individual biomarker values against age after outlier exclusion. Blue points show raw distributions; the red line depicts age‐related trend patterns.

RIs for TAT, PIC, TM, and t‐PAIC were calculated for the total population, as well as sex‐ and age‐specific groups, using the Hoffmann and Bhattacharya methods (Table 2). Figures 3 and 4 show the RI calculations for the overall population, and Figures 5 and 6 compare the sex‐ and age‐partitioned RIs. For sex differences, TAT and PIC RIs were higher in females than in males (homogeneity of variance, *F* = 0.345 and 3.505, respectively), whereas TM and t‐PAIC RIs were significantly higher in males (heterogeneity of variance, *F* = 105.195 and 17.919, respectively). All sex‐based differences were statistically significant (*p* < 0.01). For age differences, all markers showed age‐related increases in RIs except TAT, which showed no significant difference between the 0–44 and 45–59 age groups (*p* = 0.297). All other age‐group comparisons were significant (*p* < 0.01). Results are summarized in Table 2 and illustrated in Figures 6 and 7. To facilitate clinical application, quick reference summaries of the established RIs stratified by sex and by age are provided as Tables S2a and S2b, respectively.

**TABLE 2:** RIs for TAT, PIC, TM, and t‐PAIC across demographic subgroups calculated by the Hoffmann and Bhattacharya methods.

Marker	Attribute	Group	*N*	RI (Hoffmann)	RI (Bhattacharya)
TAT (ng/mL)	Total	Total	7674	0.64–3.57	0.58–2.84
	Sex	Male	4513	0.63–3.09	0.57–3.11
		Female	3161	0.63–3.94	0.59–3.91
	Age	0–44	1694	0.58–2.90	0.52–3.20
		45–59	3315	0.66–2.72	0.56–2.89
		≥ 60	2665	0.71–4.86	0.60–4.30
PIC (μg/mL)	Total	Total	7837	0.25–1.08	0.25–1.11
	Sex	Male	4579	0.25–1.00	0.23–1.06
		Female	3258	0.33–1.11	0.27–1.19
	Age	0–44	1761	0.26–0.86	0.23–0.96
		45–59	3372	0.24–0.99	0.24–1.05
		≥ 60	2704	0.28–1.40	0.28–1.31
TM (TU/mL)	Total	Total	7918	5.64–13.42	5.60–14.53
	Sex	Male	4600	5.90–13.46	5.97–13.45
		Female	3318	5.43–13.00	4.86–14.19
	Age	0–44	1787	5.32–11.35	5.18–12.22
		45–59	3397	5.74–12.55	5.89–13.28
		≥ 60	2734	6.37–14.41	6.29–14.76
t‐PAIC (ng/mL)	Total	Total	8077	3.22–15.88	3.46–15.44
	Sex	Male	4763	3.99–15.09	4.01–15.10
		Female	3314	2.87–15.04	2.81–14.89
	Age	0–44	1738	2.69–14.19	2.81–14.88
		45–59	3403	3.38–14.51	3.43–14.67
		≥ 60	2936	3.55–17.48	3.61–16.43

Abbreviations: PIC, plasmin–α2‐antiplasmin complex; RI, reference interval; TAT, thrombin–antithrombin complex; TM, thrombomodulin; t‐PAIC, tissue‐type plasminogen activator‐inhibitor complex.

### 3.4 Verification of RIs

The relative differences between the upper and lower limits of the overall population RIs for TAT, PIC, TM, and t‐PAIC established using the Hoffmann and Bhattacharya methods and those from the reagent inserts were all within the RCV range (Table 3). For PIC and TM, the RIs calculated using the Bhattacharya method were slightly wider than those calculated using the Hoffmann method, and for TM and t‐PAIC, the RIs calculated using the Hoffmann method were slightly wider than those calculated using the Bhattacharya method. RIs established using both methods passed verification, with the Bhattacharya method yielding a higher verification pass rate, as listed in Table 4.

**TABLE 3:** Comparison of overall population RIs for thrombus biomarkers from different sources and the relative differences with RCV verification.

Marker	Source	*N*	RI	Relative difference (%)	RCV (%)
TAT (ng/mL)	Reagent Insert	—	0–4.0	—	73.84
	Hoffmann method	7674	0.64–3.57	−10.75 to 16.00	
	Bhattacharya method	7674	0.58–2.84	−29.00 to 14.50	
PIC (μg/mL)	Reagent Insert	—	0–0.8	—	42.55
	Hoffmann method	7837	0.25–1.08	31.25–35.00	
	Bhattacharya method	7837	0.25–1.11	31.25–38.75	
TM (TU/mL)	Reagent Insert	—	3.8–13.3	—	55.24
	Hoffmann method	7918	5.64–13.42	0.90–48.42	
	Bhattacharya method	7918	5.60–14.53	9.24–47.36	
t‐PAIC (ng/mL)	Reagent Insert	—	0–17.00 (Male) 0–10.50 (Female)	—	53.79
	Hoffmann method	8077	3.22–15.88	−6.59 to 18.94 (Male) 30.67–51.23 (Female)	
	Bhattacharya method	8077	3.46–15.44	−9.18 to 20.35 (Male) 32.95–47.05 (Female)	

*Note:* The manufacturer‐provided RIs (reagent insert) for all four markers were obtained from Sysmex Corporation (Kobe, Japan) and were established using 134 healthy Japanese individuals. The age distribution of the reference population was not specified by the manufacturer.

Abbreviations: RCV, reference change value; RI, reference interval.

**TABLE 4:** Verification pass rates of Hoffmann and Bhattacharya method‐derived RIs for the thrombus markers.

Marker	Group	Hoffmann method		Bhattacharya method	
		RI	Verification pass rate (%)	RI	Verification pass rate (%)
TAT (ng/mL)	Total	0.64–3.57	100	0.58–2.84	100
	Male	0.63–3.09	100	0.57–3.11	100
	Female	0.63–3.94	100	0.59–3.91	100
	0–44	0.58–2.90	95	0.52–3.20	100
	45–59	0.66–2.72	100	0.56–2.89	100
	≥ 60	0.71–4.86	100	0.60–4.30	100
PIC (μg/mL)	Total	0.25–1.08	100	0.25–1.11	100
	Male	0.25–1.00	100	0.23–1.06	100
	Female	0.33–1.11	100	0.27–1.19	100
	0–44	0.26–0.86	90	0.23–0.96	95
	45–59	0.24–0.99	100	0.24–1.05	100
	≥ 60	0.28–1.40	100	0.28–1.31	100
TM (TU/mL)	Total	5.64–13.42	100	5.60–14.53	100
	Male	5.90–13.46	100	5.97–13.45	100
	Female	5.43–13.00	100	4.86–14.19	100
	0–44	5.32–11.35	100	5.18–12.22	100
	45–59	5.74–12.55	100	5.89–13.28	100
	≥ 60	6.37–14.41	95	6.29–14.76	100
t‐PAIC (ng/mL)	Total	3.22–15.88	100	3.46–15.44	100
	Male	3.99–15.09	100	4.01–15.10	100
	Female	2.87–15.04	100	2.81–14.89	100
	0–44	2.69–14.19	100	2.81–14.88	100
	45–59	3.38–14.51	100	3.43–14.67	100
	≥ 60	3.55–17.48	100	3.61–16.43	100

## 4 Discussion

VTE is a serious clinical condition that increases the risk of complications such as stroke and heart disease and imposes a substantial burden on families and healthcare systems [28]. Thrombus formation involves complex interactions among the vascular endothelium, coagulation, and fibrinolytic systems. Although anticoagulant and thrombolytic therapies exist, individualized assessment of their efficacy remains limited.

TAT, PIC, TM, and t‐PAIC are novel biomarkers reflecting early activation of coagulation and fibrinolysis as well as vascular endothelial injury. They have demonstrated significant value in disease diagnosis and treatment monitoring [12, 13]. Studies have reported that TAT and PIC levels rise significantly in patients with DIC before and after onset [29], and their ratio can serve as a prognostic indicator [30]. Accurate monitoring of these markers is therefore crucial for evaluating VTE risk and treatment effectiveness. Reliable laboratory RIs are fundamental for precise clinical assessment, and laboratories should establish and regularly review population‐specific RIs [31]. Currently, most laboratories rely on reagent insert RIs based on Western populations, while indirect methods are increasingly recommended by the International Federation of Clinical Chemistry (IFCC). This study is the first to use indirect methods on large clinical datasets to establish personalized RIs for these markers in a Chinese population, providing practical guidance for clinical diagnosis and treatment.

Our results show that all four markers increase with age. TAT and PIC levels were higher in females, whereas TM and t‐PAIC were higher in males. The indirect method, based on large‐scale clinical data across a continuous age range, captures population‐ level trends and includes individuals with subclinical hypercoagulability or endothelial dysfunction. Therefore, the observed age‐related increases likely reflect a genuine association between aging and enhanced coagulation‐fibrinolysis activity. This may reflect age‐related vascular endothelial dysfunction, chronic inflammation, reduced anticoagulant factors, and altered fibrinolytic activity, collectively promoting coagulation activation [32, 33, 34, 35, 36]. Additionally, the majority of Chinese women experience menopause between 45 and 55 years, and the postmenopausal decline in estrogen reduces vascular protection and increases metabolic syndrome risk, promoting proinflammatory and procoagulant states [37]. This may partly explain the higher TAT and PIC levels in females. Moreover, chronic inflammatory conditions such as autoimmune diseases have a higher prevalence in women and are often accompanied by persistent, low‐grade coagulation activation [38], further contributing to higher TAT and PIC levels in females. TM, which captures thrombin, is consumed in complex form during coagulation activation, potentially explaining its lower levels in females. t‐PAIC, formed by the 1:1 binding of PAI‐1 to t‐PA, reflects inhibition of fibrinolysis [39, 40], and its higher levels in males may be associated with metabolic syndrome‐related endothelial dysfunction (e.g., hypertension, insulin resistance) [41].

When comparing indirect method‐derived RIs with the manufacturer's insert values, the largest relative difference was observed for the upper limit of the female t‐PAIC RI (51.23%). t‐PAIC levels are influenced by genetic, hormonal, and metabolic factors. East Asian populations differ from Western populations in PAI‐1 gene polymorphisms, which may lead to different baseline t‐PAIC levels [42]. In addition, methodological differences between detection systems may also contribute. Although relatively large, this difference remains within the RCV range, indicating that it likely arises from population characteristics and analytical variability rather than analytical error alone. This highlights the importance of establishing population‐specific RIs.

We also compared indirect method results for the ≥ 60‐year subgroup with those from our previous direct method study in healthy older adults using the Wondfo chemiluminescence platform [43]. Although the two platforms differed, the comparison focused on age‐and sex‐related trends rather than absolute values. The direct method yielded lower RI limits (TAT: 0.51–3.72 ng/mL, PIC: 0.10–1.04 μg/mL, TM: 2.96–13.26 TU/mL, t‐PAIC: 1.63–11.34 ng/mL) reflecting its basis in strictly screened healthy individuals, whereas the indirect method includes individuals with subclinical abnormalities [44, 45]. Thus, the slightly wider RIs from the indirect method reflect broader real‐world distributions rather than methodological inferiority. Importantly, both methods showed consistent age‐and sex‐related trends (e.g., TAT and PIC increase with age, TAT higher in females), mutually validating the reliability of the established RIs.

Although some studies have used screened or health examination populations to establish RIs [16, 46], real‐world clinical data offer greater practicality. Indirect methods can reliably establish RIs even when 10%–30% of the population has abnormal values [19, 25]. In this study, both Hoffmann and Bhattacharya methods produced consistent results, verified by RCV and high validation pass rates. The Bhattacharya method showed slightly higher pass rates in some subgroups, possibly due to its flexibility in handling complex distributions commonly seen in real‐world clinical data [47, 48]. However, both methods met the CLSI acceptance criterion (> 90%), indicating comparable performance. Future studies should incorporate clinical feedback or direct‐method comparisons to determine the optimal implementation.

By analyzing large‐scale data, this study established sex‐ and age‐specific RIs for thrombus markers, supporting early screening and diagnosis of thrombotic diseases [49, 50] and individualized treatment decisions. In the era of precision medicine, these RIs may improve thrombosis risk assessment, guide refined intervention strategies, and improve patient outcomes [49].

However, the study has limitations. First, its single‐center design and sample size may limit generalizability. Second, the absence of long‐term follow‐up restricted insights into dynamic marker changes during disease progression. Third, although sex and age were identified as significant determinants of all four markers, RIs were established separately for each factor rather than for sex‐by‐age subgroups, which may limit precision for specific demographic combinations. Future multicenter studies with larger sample sizes and long‐term follow‐up are needed to validate these RIs and enhance their clinical applicability.

## 5 Conclusions

This study established and validated comprehensive RIs for four thrombus biomarkers (TAT, PIC, TM, and t‐PAIC) in a Chinese population using Hoffmann and Bhattacharya indirect methods. Significant demographic variations were observed: TAT and PIC levels were higher in females, while TM and t‐PAIC levels were higher in males, and all markers increased with age. These findings underscore the necessity for population‐specific, sex‐ and age‐stratified RIs, providing a practical tool for early detection and risk assessment of thrombotic diseases, supporting more precise clinical decision‐making.

## Author Contributions

Lei Zhang acquired the data, drafted the initial manuscript, reviewed and revised the manuscript. Qiyu Hu analyzed all data. Chen Yiming drew the figures and tables of the article. Hui Yuan took charge of the design of the research scheme and gave final approval of the version to be published. All authors read and approved the final manuscript.

## Funding

This study was financially supported by Beijing Municipal Administration of Hospitals Incubating Program (Grant PX2025023).

## Ethics Statement

This study was approved by the Ethics Committee of Beijing Anzhen Hospital, the affiliated hospital of Capital Medical University (2024306X).

## Conflicts of Interest

The authors declare no competing interests. All instruments and reagents used in this study were purchased from commercial sources (Sysmex Corporation, Kobe, Japan) and were operated according to the manufacturer's instructions. The authors have no financial or personal relationships with the manufacturer that could inappropriately influence this work.

## Supporting information


**Table S1:** Batch numbers of reagents, calibrators, and quality controls for the four thrombus markers.
**Table S2a:** Sex‐stratified RIs for thrombus biomarkers in a Chinese population (Bhattacharya method).
**Table S2b:** Age‐stratified reference intervals for thrombus biomarkers in a Chinese population (Bhattacharya method).

## Data Availability

The datasets generated during and/or analyzed during the current study are available from the corresponding author on reasonable request.
