# Supplementary material for: Establishment of Reference Intervals for Thrombus Biomarkers in a Single‐Center Chinese Population Using Indirect Methods
Source: J Clin Lab Anal. 2026 Sep 9;40(18):e70315. doi: 10.1002/jcla.70315 (PMC13555359; doi:10.1002/jcla.70315)
Supplement: Supplementary file 1 [file JCLA-40-e70315-s001.docx]

**Supplementary Table 1.** Batch Numbers of Reagents, Calibrators, and Quality Controls for the Four Thrombus Markers

| Marker | TAT | PIC | TM | t-PAIC |
| --- | --- | --- | --- | --- |
| Reagent  Batch Numbers | TJ0761、UB0801 UF0821、VL0931 | TI0681、TJ0691 UC0721、VL0811 | UA0731、UH0781 VB0831、WC0901 | TB0671、UA0701 UE0731、WB0821 |
| Calibrator Batch Numbers | OK0281、QA0341 UB0571 | PF0281、QG0341 UB0551 | QC0321、RE0341 TK0471 | QB0311、QH0341 SF0441 |
| QC  Batch Numbers | QTA-013、QTA-019 QTA-023、QTA-051 | QPI-015、QPI-017 QPI-019、QPI-021 | QTM-016、QTM-022 QTM-024、QTM-028 | QTP-015、QTP-017 QTP-020、QTP-027 |

Note: All reagents, calibrators, and quality controls were sourced from Sysmex Corporation (Kobe, Japan). Multiple batches were used throughout the study period (2020–2024), and all lots met the manufacturer’s quality specifications.

**Supplementary Table 2a.** Sex-stratified RIs for Thrombus Biomarkers in a Chinese Population (Bhattacharya Method)

| Marker | Sex Group | RI |
| --- | --- | --- |
| TAT (ng/mL) | Male | 0.57–3.11 |
|  | Female | 0.59–3.91 |
| PIC (μg/mL) | Male | 0.23–1.06 |
|  | Female | 0.27–1.19 |
| TM (TU/mL) | Male | 5.97–13.45 |
|  | Female | 4.86–14.19 |
| t-PAIC (ng/mL) | Male | 4.01–15.10 |
|  | Female | 2.81–14.89 |

**Supplementary Table 2b.** Age-stratified Reference Intervals for Thrombus Biomarkers in a Chinese Population (Bhattacharya Method)

| Marker | Age Group | RI |
| --- | --- | --- |
| TAT (ng/mL) | 0–44 | 0.52–3.20 |
|  | 45–59 | 0.56–2.89 |
|  | ≥60 | 0.60–4.30 |
| PIC (μg/mL) | 0–44 | 0.23–0.96 |
|  | 45–59 | 0.24–1.05 |
|  | ≥60 | 0.28–1.31 |
| TM (TU/mL) | 0–44 | 5.18–12.22 |
|  | 45–59 | 5.89–13.28 |
|  | ≥60 | 6.29–14.76 |
| t-PAIC (ng/mL) | 0–44 | 2.81–14.88 |
|  | 45–59 | 3.43–14.67 |
|  | ≥60 | 3.61–16.43 |

Note: Sex and age were analyzed as independent stratification factors; therefore, sex-by-age subgroups are not provided. For clinical use, we suggest prioritizing the stratification factor with greater physiological relevance based on the specific marker: sex-stratified RIs may be preferred for TAT and PIC (higher in females) and for TM and t-PAIC (higher in males), while age-stratified RIs are recommended for elderly patients (≥60 years) given the consistent age-related increases across all markers. For intervals derived by the Hoffmann method and for the overall population, refer to Table 2 in the main text. All intervals represent the central 95% range (2.5th–97.5th percentiles).
